# Multimodality Imaging of Caval and Coronary Sinus Venous Anomalies

**DOI:** 10.1016/j.case.2022.06.007

**Published:** 2022-08-13

**Authors:** Jordan Liebman, Daniel Bamira, Richard Ro, Alan F. Vainrib, Adam J. Small, Robert Donnino, Muhamed Saric

**Affiliations:** a Leon H. Charney Division of Cardiology, NYU Grossman School of Medicine, NYU Langone Health, New York, New York; b Department of Radiology, NYU Grossman School of Medicine, NYU Langone Health, New York, New York; c Veterans Affairs Medical Center, Manhattan Campus, New York, New York

**Keywords:** Venous anomalies, Superior vena cava, Coronary sinus, Echocardiography, Agitated saline

## Abstract

• Abnormal fetal development can produce several anomalies of the caval venous system.
• We present multimodality imaging of the most common caval venous anomalies.
• Each imaging modality provides incremental value when identifying these anomalies.
• Even normal variants may impact pacing lead or central venous catheter placement.
• Pathologic variants may lead to intracardiac shunting.

## Graphical abstract

**Figure:**
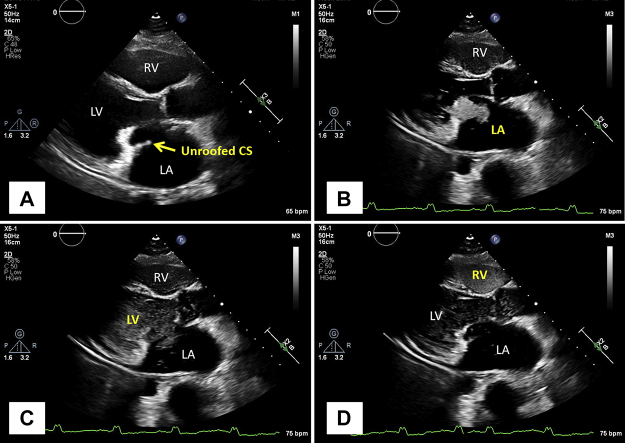

## Introduction

By 5 weeks of gestation, 3 distinct venous systems begin to develop within the growing embryo: the cardinal, umbilical, and vitelline veins. In this case series, we discuss anomalies arising from maldevelopment of the cardinal system.^1^^,^^2^

The cardinal veins, the precursors of the caval venous system, are divided into pairs of anterior and posterior cardinal veins, draining the cranial and caudal parts of the embryo, respectively. On either side of the embryo, anterior and posterior cardinal veins merge into a common cardinal vein and drain intraembryonic blood to the primitive atrium through the sinus venosus.

Although the cardinal venous system initially develops symmetrically, normal development dictates that the left side regresses to form the lateralized venous structures recognizable in mature humans. Over the next several weeks of development, the left anterior cardinal vein extends an anastomotic segment toward the right anterior cardinal vein, forming what will become the left brachiocephalic (innominate) vein. Cranial to this anastomosis, the left anterior cardinal vein forms the left internal jugular vein. Caudal to the anastomosis, the left anterior cardinal vein typically regresses in its proximal portion but remains patent in its distal portion to form the oblique vein (vein of Marshall), the great cardiac vein, and the coronary sinus. On the right side, the cranial part of the right anterior cardinal vein becomes the right internal jugular vein, while the caudal part, along with the right common cardinal vein and right horn of the sinus venosus, forms the superior vena cava (SVC).

Given the complexities of the cardinal venous system development, which includes lateralization, anastomoses, and involutions, multiple patterns of venous drainage to the heart from the upper body may arise. The typical pattern includes a brachiocephalic vein draining the venous blood from the left arm and the left side of the head into a right-sided SVC and then into the right atrium (RA). Simultaneously, the vein of Marshall, the great cardiac vein, and the coronary sinus lose any connection to the caval system and drain the venous blood of the heart itself into the RA.

Here we present 5 cases of anomalous venous patterns of the cardinal system.

## Case Presentation 1

**Figure 1:**
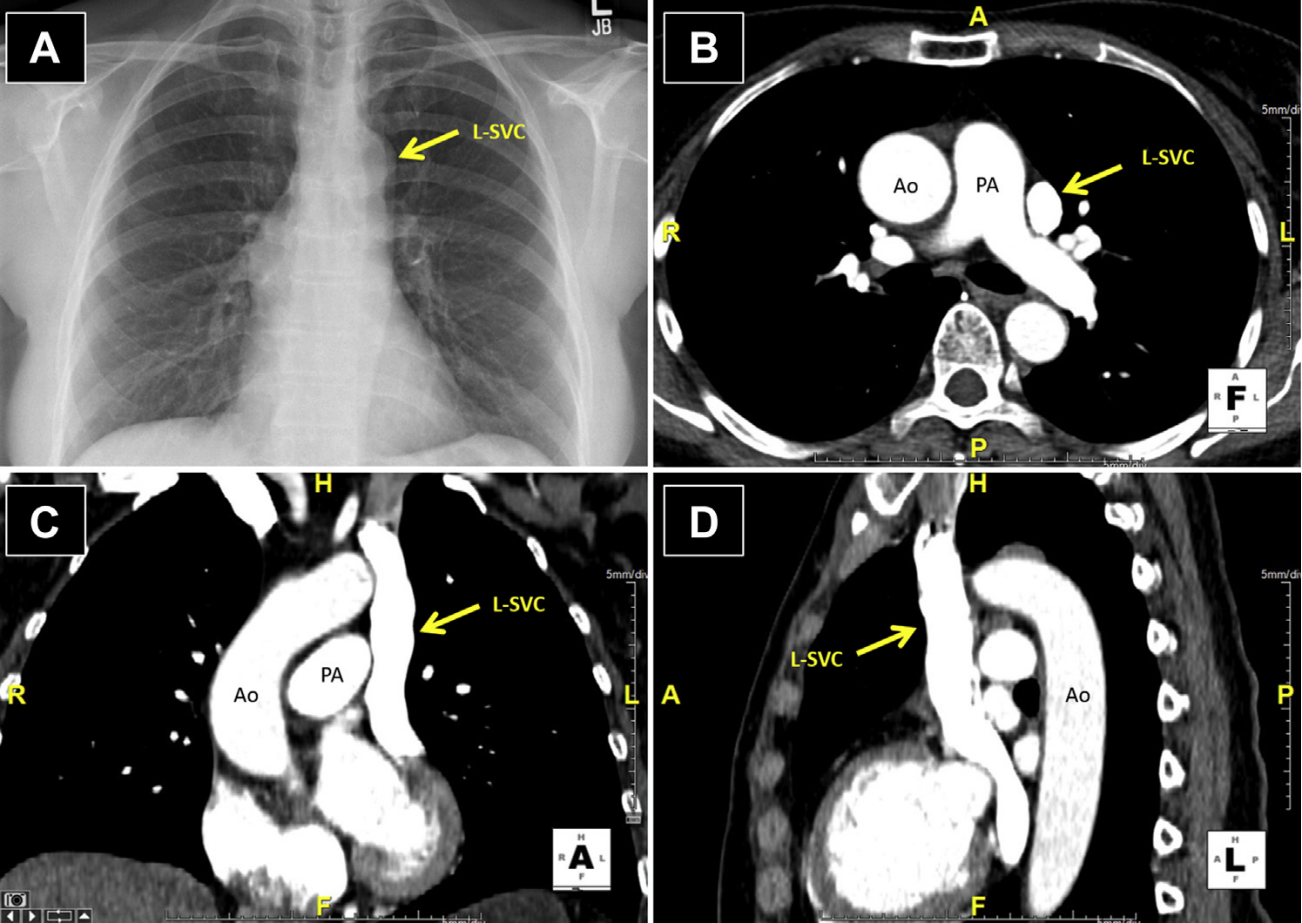
Case 1: persistent L-SVC. Chest x-ray with anteroposterior projection and CCT with axial, coronal, and sagittal views. A widened mediastinum on chest x-ray **(A)** was initially concerning for aortic dissection (*arrow*), but subsequent CCT imaging in this patient identified it as a persistent L-SVC **(B-D)**. *Ao*, Aorta; *PA*, pulmonary artery.

A 62-year-old woman with a history of a childhood murmur, anxiety, and ovarian cysts presented to an urgent care clinic with palpitations. She denied any changes in exercise tolerance, orthopnea, or paroxysmal nocturnal dyspnea. A chest x-ray at the clinic revealed a widened mediastinum concerning for aortic dissection (Figure 1A). Subsequent cardiac computed tomography (CCT) demonstrated a normal ascending aorta and a persistent left SVC (L-SVC) draining into an intact coronary sinus (Figure 1B-D).

**Figure 2:**
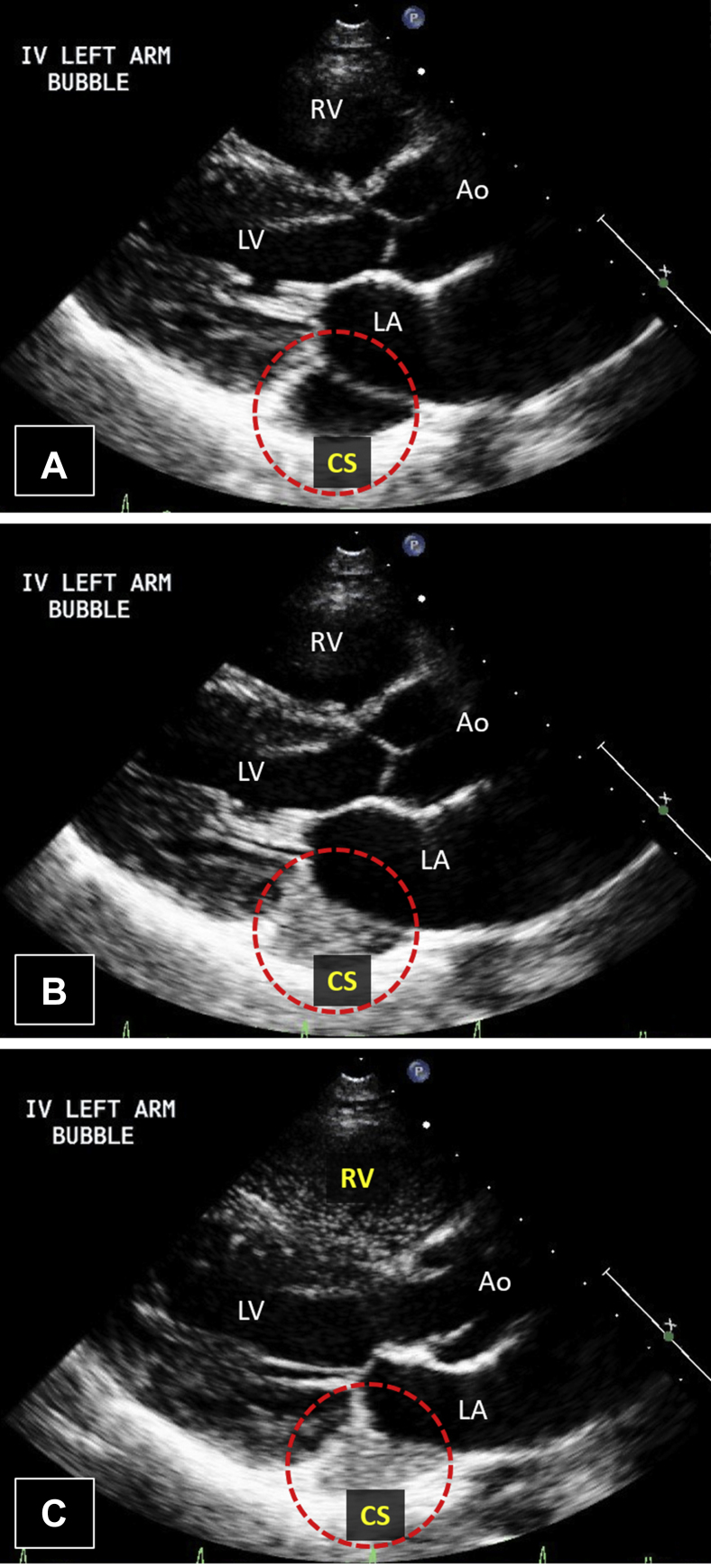
Case 1: persistent L-SVC. Transthoracic echocardiogram in parasternal long-axis view with intravenous agitated saline injection in left arm. The presence of a dilated CS on TTE **(A)** suggests a venous anomaly. After injection of agitated saline into the patient's left arm, the dilated CS opacifies first **(B)**, followed by the right heart **(C)**. This opacification pattern is consistent with the diagnosis of a persistent L-SVC. Video 1 corresponds to this figure. *Ao*, Aorta; *CS*, coronary sinus; *RV*, right ventricle.

Transthoracic echocardiogram (TTE) was remarkable only for a markedly dilated coronary sinus. After intravenous injection of agitated saline in the left arm, the pattern of opacification (coronary sinus first, right heart next) was consistent with a persistent L-SVC (Figure 2, Video 1). After intravenous injection of agitated saline in the right arm, there was opacification of the right heart only. Overall, these findings were consistent with a persistent L-SVC in the presence of a normal right SVC (R-SVC).

This case demonstrates the typical scenario in which a persistent L-SVC is diagnosed incidentally by CCT, TTE, or other imaging modality. As is typical for an uncomplicated persistent L-SVC, there was no shunt, and the finding in her is considered a normal variant rather than a pathologic anomaly.

## Case Presentation 2

A 40-year-old man with a history of untreated hypertension presented to a cardiologist for evaluation of new-onset substernal chest pain lasting several weeks.

**Figure 3:**
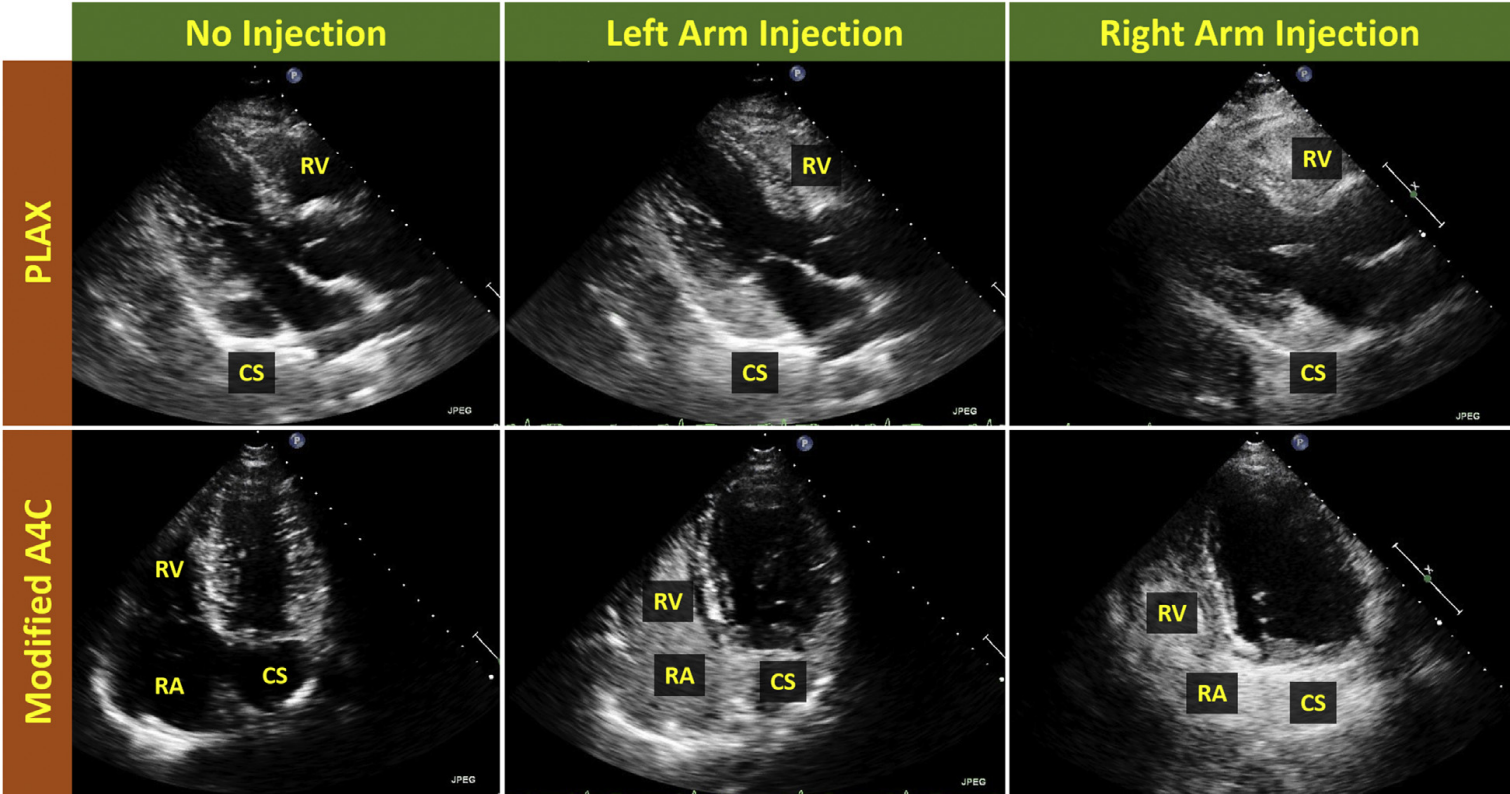
Case 2: persistent L-SVC, absent R-SVC. Transthoracic echocardiogram in PLAX and modified A4C views with intravenous agitated saline injection in left and right arms. After left arm agitated saline injection, the pattern of opacification (dilated CS first, right heart next) is consistent with the diagnosis of a persistent L-SVC and is similar to the opacification pattern seen in Figure 2. After right arm agitated saline injection, the pattern of opacification is the same as after left arm injection. This suggests the absence of an R-SVC. Videos 2 and 3 correspond to this figure. *A4C*, Apical four-chamber; *CS*, coronary sinus; *PLAX*, parasternal long axis; *RV*, right ventricle.

Transthoracic echocardiogram was remarkable only for a markedly dilated coronary sinus. After intravenous injection of agitated saline in the left arm, the pattern of opacification (coronary sinus first, right heart next) was consistent with a persistent L-SVC (Figure 3, Video 2). After intravenous injection of agitated saline in the right arm, the same pattern of opacification (coronary sinus first, right heart next) was observed (Figure 3, Video 3). These findings were consistent with a persistent L-SVC and absent R-SVC.

This venous pattern can also be considered a normal variant rather than a true pathologic finding as there is no shunt, just an alternative venous pathway to the heart.

## Case Presentation 3

**Figure 4:**
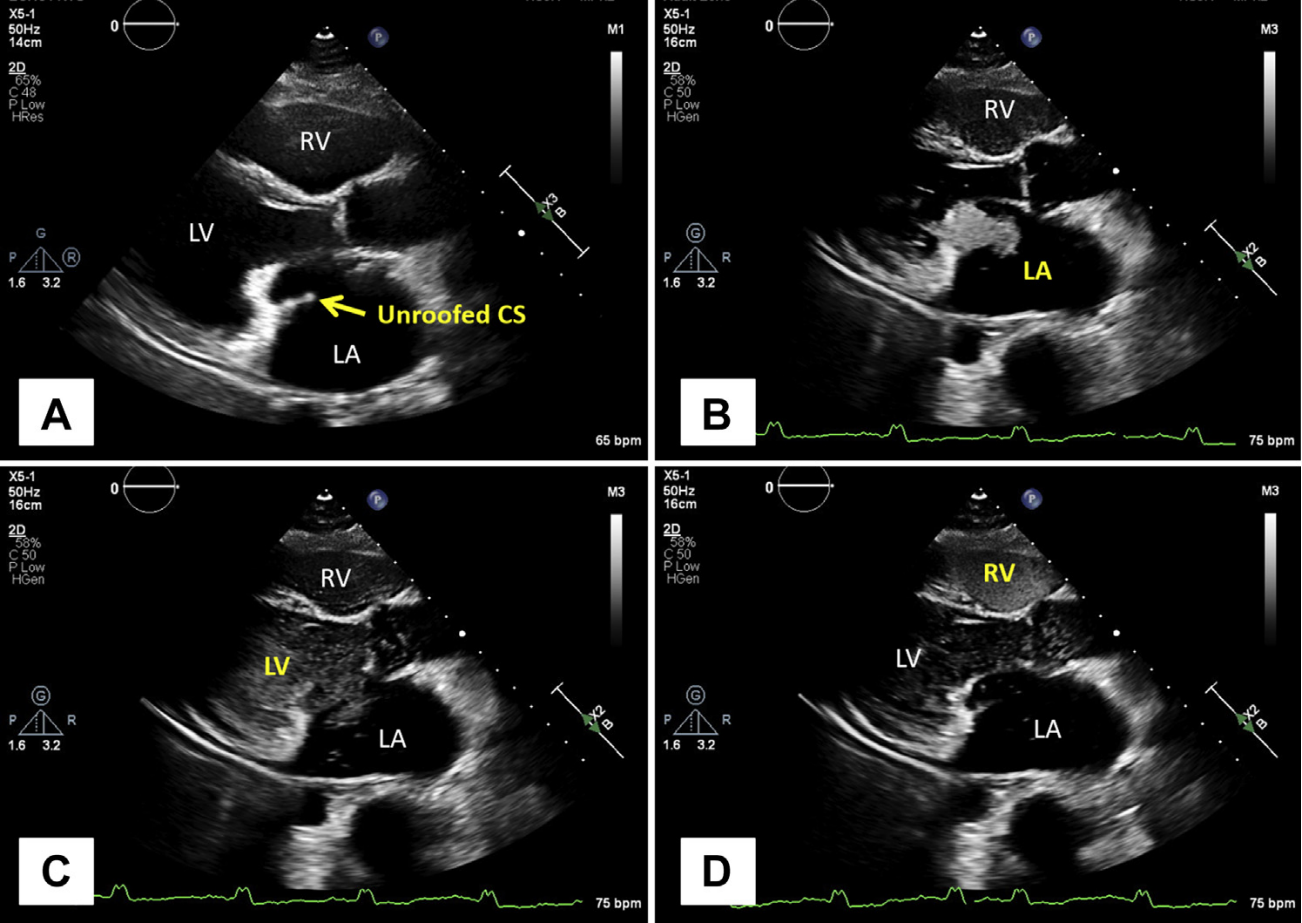
Case 3: persistent L-SVC, unroofed CS. Transthoracic echocardiogram in parasternal long-axis view with intravenous agitated saline injection in the left arm. The continuity between the CS and LA **(A)** confirms the presence of an unroofed CS. After injection of agitated saline into the patient's left arm, the CS opacifies first **(B)**, followed by the left heart **(C)** and the right heart **(D)**. The opacification of the CS first is diagnostic of an L-SVC, while the opacification of the left heart before the right heart confirms the unroofed CS. The co-occurrence of these 2 findings is also known as Raghib syndrome. Video 4 corresponds to this figure. *CS*, Coronary sinus; *RV*, right ventricle.

A 59-year-old woman with a history of atrial flutter and a remote primum atrial septal defect (ASD) and mitral valve repair surgery presented with palpitations and shortness of breath. Electrocardiogram showed atrial flutter with 2:1 atrioventricular conduction and a ventricular rate of 120 beats per minute. Transthoracic echocardiogram was remarkable for a severely dilated left atrium (LA) and an unusual linear echogenic structure along the plane of the mitral valve seemingly arising from the posterior mitral annulus. After intravenous injection of agitated saline in the left arm, the pattern of opacification was as follows: coronary sinus first and LA next, followed by the left ventricle (LV) and right heart. The findings were consistent with a combination of a persistent L-SVC and an unroofed coronary sinus (Figure 4, Video 4).

**Figure 5:**
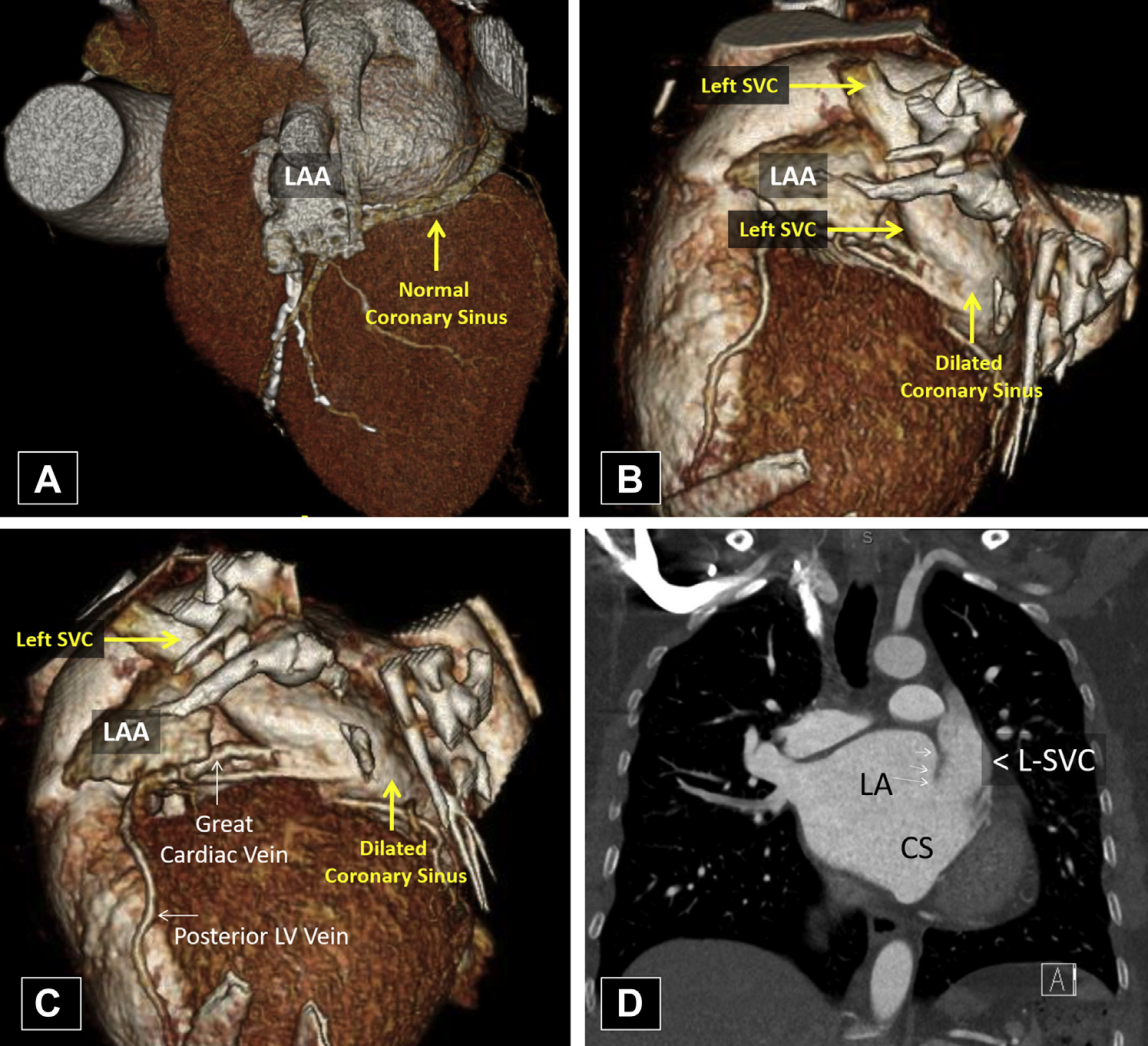
Case 3: persistent L-SVC, unroofed CS. Cardiac CT with three-dimensional reconstruction **(A-C)** and coronal section **(D)**. This patient's CS **(B, C)** is markedly dilated when contrasted with a normal CS **(A)**. This is the result of an atypical connection with an L-SVC **(B-D)** that drains venous blood from the upper body and great cardiac vein via the CS. In addition, this patient's CS appears continuous with the LA **(D)**, indicative of an unroofed CS. This produces a direct connection between the venous system and the left heart and a right-to-left shunt. *CS*, Coronary sinus; *LAA*, left atrial appendage.

Cardiac computed tomography (CT; Figure 5) and cardiovascular magnetic resonance imaging (CMR) revealed bilateral SVCs with the L-SVC draining directly into the LA without a discrete coronary sinus, indicative of a completely unroofed coronary sinus resulting in a right-to-left shunt. The great cardiac vein was seen draining into the L-SVC. The R-SVC drained normally into the RA, and there was a small bridging vein connecting the L-SVC and R-SVC. Additionally, a prominent membrane crossing the lateral aspect of the LA was visualized, suggestive of nonobstructive cor triatriatum. The overall findings were consistent with the so-called Raghib syndrome. The patient declined surgical repair.

## Case Presentation 4

An 84-year-old woman presented with fatigue and shortness of breath. Transthoracic echocardiogram demonstrated severe high-gradient aortic stenosis with preserved left ventricular ejection fraction. She was then referred for evaluation for possible transcatheter aortic valve implantation (TAVI).

**Figure 6:**
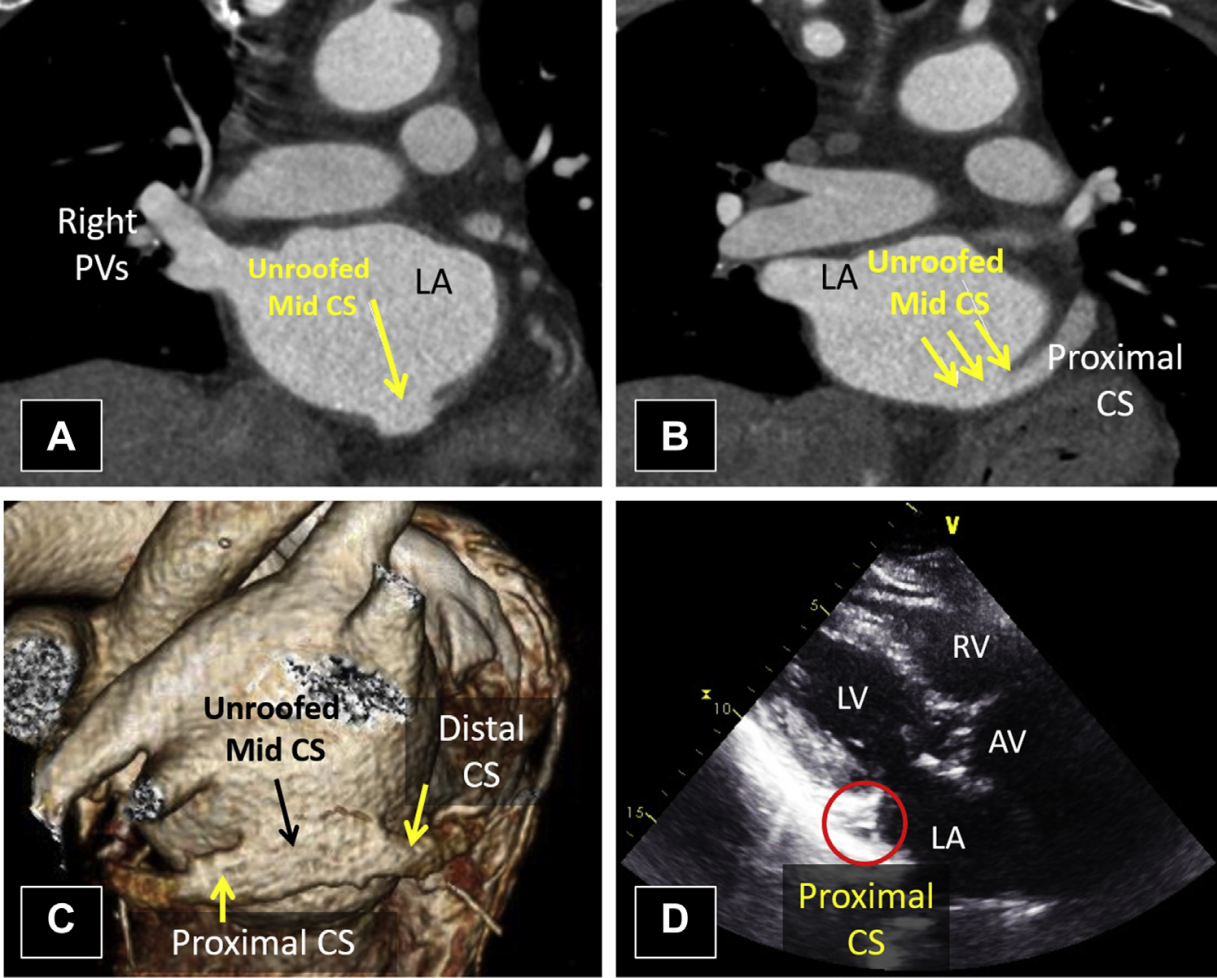
Case 4: unroofed CS without persistent L-SVC. Cardiac CT imaging with coronal sections **(A, B)** and three-dimensional rendering **(C)** and TTE in the parasternal long-axis view. The lack of a discrete division between the LA and the middle segment of the CS indicates an unroofed mid CS **(A-C)**. While the connection between the CS and LA is seen on CCT imaging, the CS appears normal and nondilated on TTE **(D)**. This is because TTE visualized the proximal segment of the CS. Video 5 corresponds to this figure. *AV*, Aortic valve; *CS*, coronary sinus; *PV*, pulmonary vein; *RV*, right ventricle.

Routine pre-TAVI CCT revealed a markedly calcified and severely stenotic trileaflet aortic valve. There was also an incidental finding of an unroofed mid coronary sinus (Figure 6A-C) with a stenotic connection to the RA, limiting left-to-right intracardiac shunting. Additionally, the oxyhemoglobin saturation ranged from 94% to 97%, suggesting minimal net right-to-left shunting. Interestingly, no coronary sinus abnormality could be visualized by TTE (Figure 6D, Video 5).

The patient underwent an uncomplicated TAVI procedure. Surgical repair of the unroofed coronary sinus with absent L-SVC was deemed unnecessary.

## Case Presentation 5

**Figure 7:**
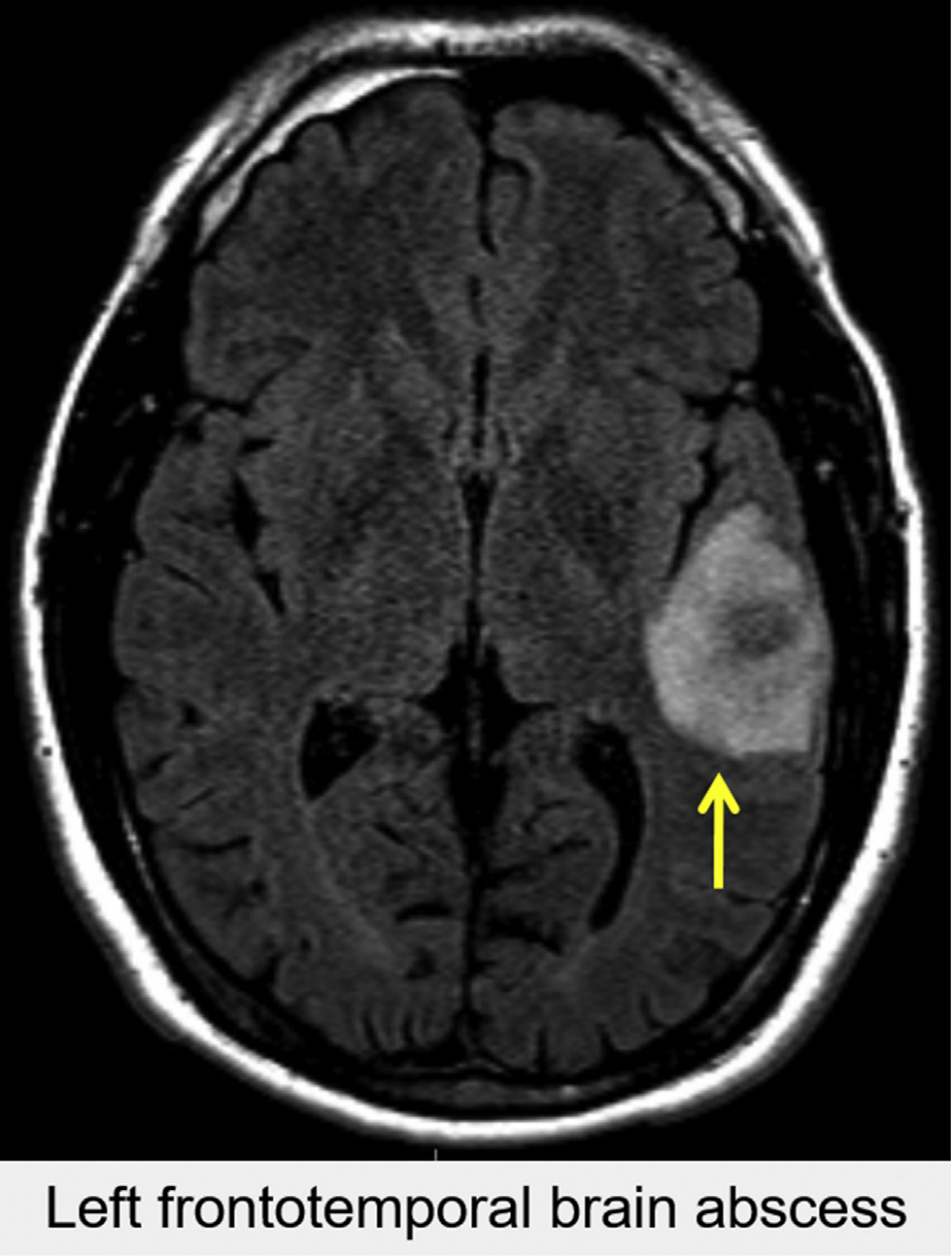
Case 5: R-SVC draining into the LA. Axial noncontrast head CT identified a round hyperdense lesion with a necrotic center located in the left temporal region of the patient's brain. This patient's clinical history and the appearance of the lesion is consistent with a brain abscess.

A 36-year-old man with no medical history presented following a new-onset seizure. Three weeks prior, he had handled several items with dog urine while cleaning a tenant's apartment. After noncontrast head CT (Figure 7) and brain magnetic resonance imaging demonstrated a left temporal lobe abscess, the patient underwent craniotomy and abscess drainage. Intraoperative cultures grew *Capnocytophagia cani* and *Streptococcus intermedius*. Postoperative transesophageal echocardiogram (TEE) revealed no intracardiac vegetation, thrombi, or masses.

**Figure 8:**
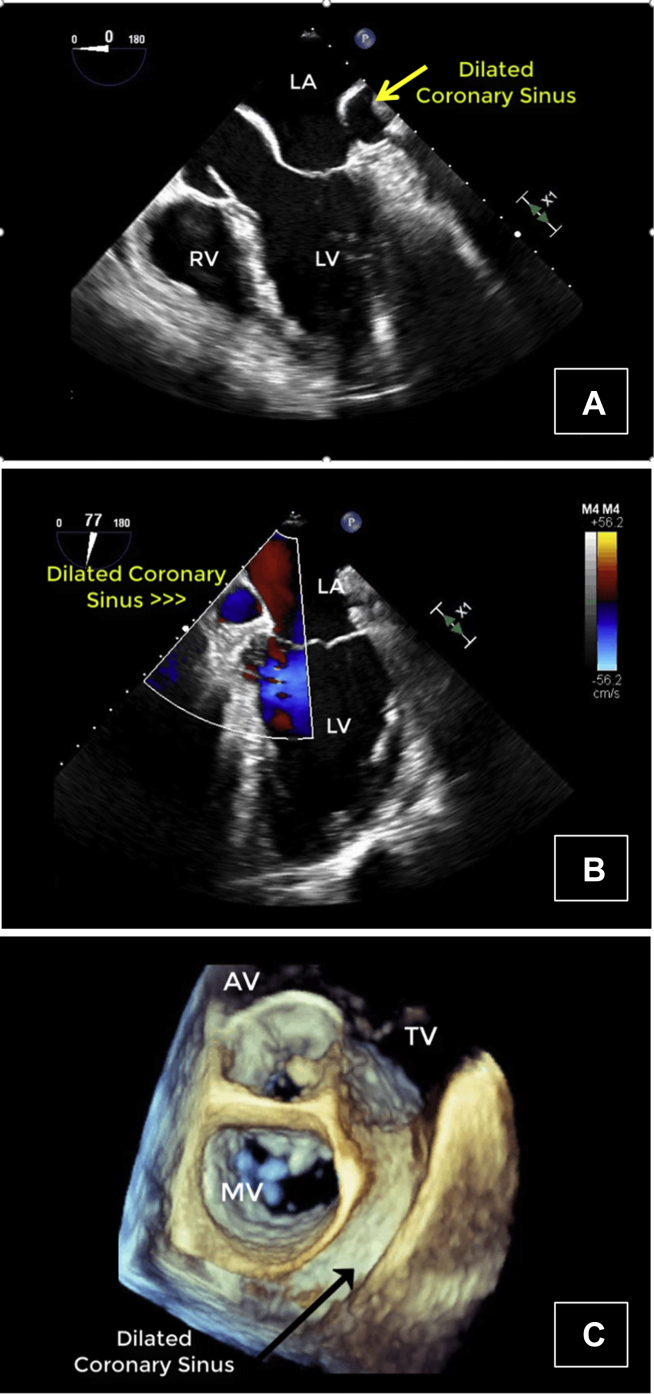
Case 5: R-SVC draining into the LA. Transesophageal echocardiogram with midesophageal view at 0° in grayscale **(A)**, midesophageal view at 77° with color Doppler **(B)**, and a three-dimensional zoom image in the so-called surgical view of the mitral valve **(C)**. Transesophageal echocardiogram demonstrated a markedly dilated coronary sinus **(A-C)** with significant blood flow **(B)**. As shown in other cases, a dilated coronary sinus suggests an atypical pattern of venous drainage and is usually consistent with diagnosis of a persistent L-SVC. Videos 6 through 8 correspond to this figure. *AV*, Aortic valve; *MV*, mitral valve; *RV*, right ventricle; *TV*, tricuspid valve.

**Figure 9:**
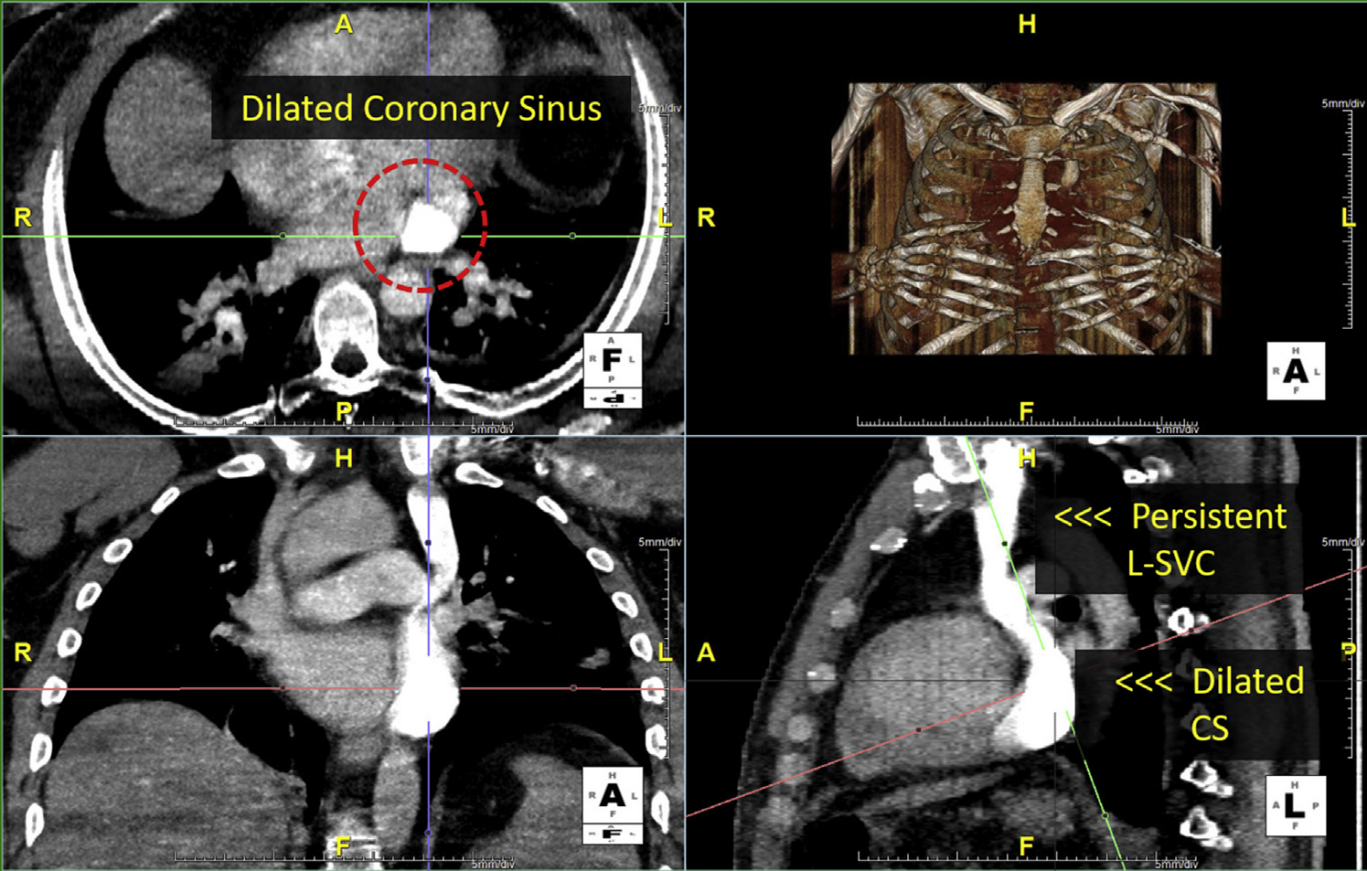
Case 5: R-SVC draining into the LA. Cardiac CT with axial, coronal, and sagittal views. Subsequent CCT imaging with contrast identified a persistent L-SVC draining into a notably dilated CS. The dilated CS is located at the intersection of cross hairs. The identification of an L-SVC on CCT imaging confirms the suspected diagnosis of an L-SVC based on the dilated CS seen on TEE. *CS*, Coronary sinus.

**Figure 10:**
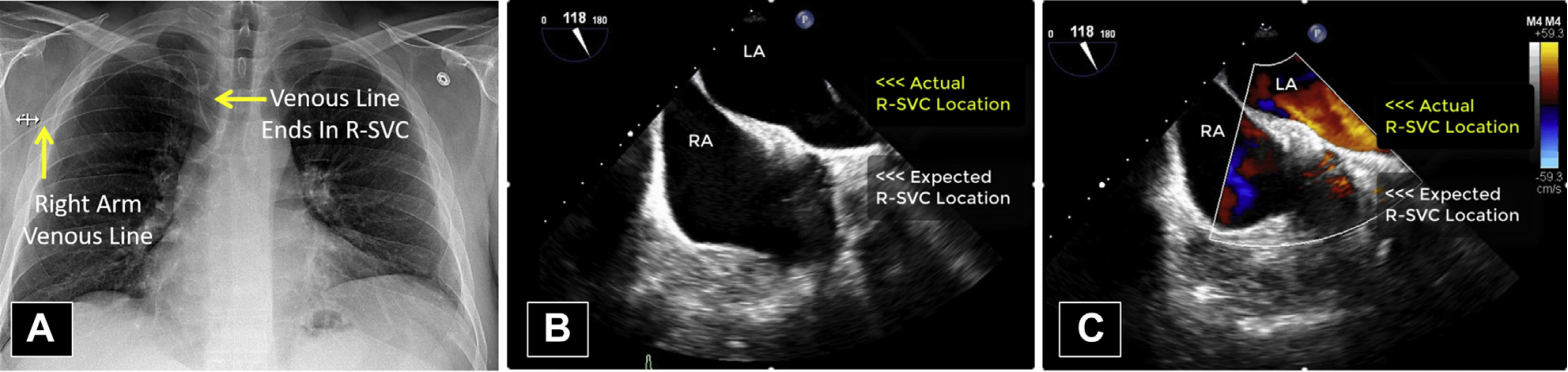
Case 5: R-SVC draining into the LA. Chest x-ray with anteroposterior projection **(A)** and TEE with midesophageal view at 118° in grayscale **(B)** and with color Doppler **(C)**. A chest x-ray **(A)** taken to confirm the position of a right arm venous line confirms the presence of an R-SVC. However, TEE **(B, C)** reveals that the R-SVC in this patient is not in the usual location; instead, it is located further posterior where it drains into the LA. Videos 9 and 10 correspond to this figure.

**Figure 11:**
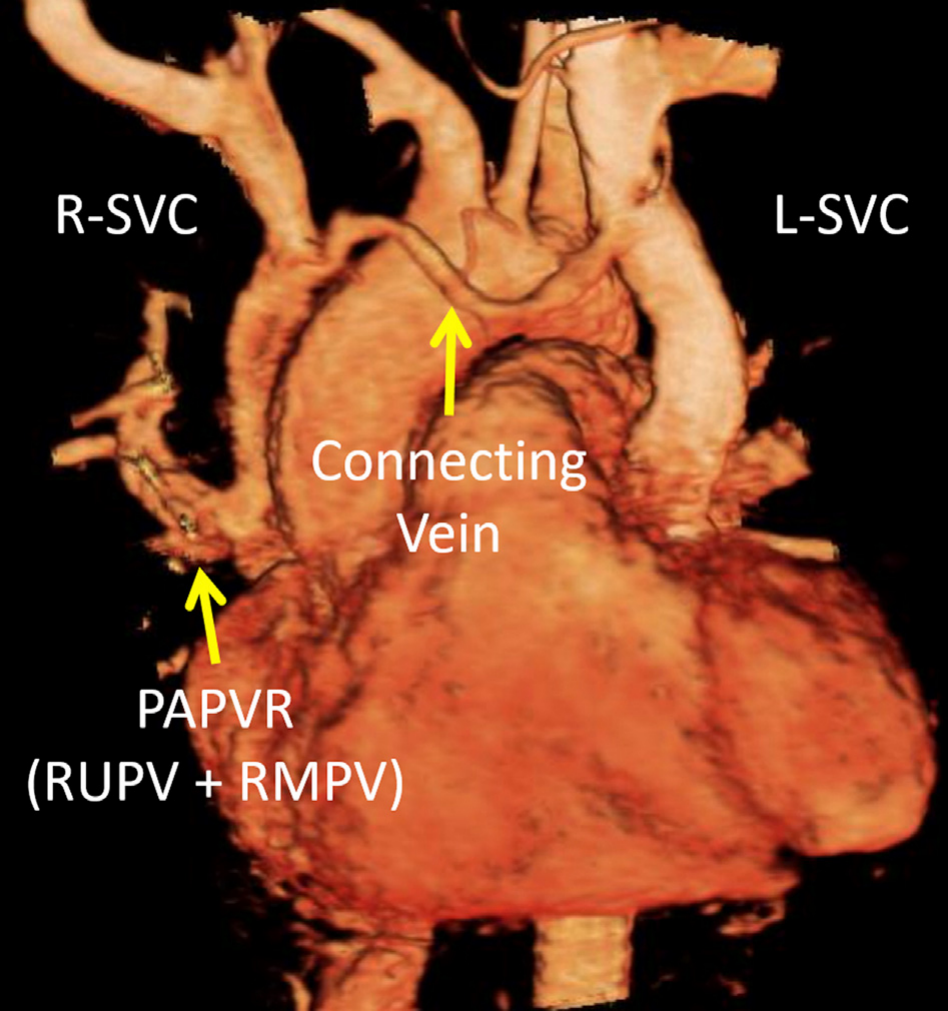
Case 5: R-SVC draining into the LA. Three-dimensional rendered CMR, which helps further characterize this patient's atypical venous anatomy. There is a small connecting vein between the R-SVC and L-SVC as well as 2 pulmonary veins draining into the R-SVC. The PAPVR is unrelated to the presence of a persistent L-SVC, since the former does not derive from the embryological cardinal venous system. Still, the presence of several anomalous venous structures and connections creates the potential for a highly unusual pattern of venous drainage. *RMPV*, Right middle pulmonary vein; *RUPV*, right upper pulmonary vein.

However, there were several vascular anomalies, including a persistent L-SVC draining into a dilated coronary sinus and then into the RA, visualized by TEE (Figure 8, Videos 6-8) and CCT (Figure 9). There was an R-SVC overriding a sinus venosus defect, so that the R-SVC drained primarily into the LA (Figure 10, Videos 9 and 10). The sinus venosus defect was associated with partial anomalous pulmonary venous return (PAPVR), as the right superior pulmonary vein was visualized draining into the R-SVC. There was also a small bridging vein visualized by CMR connecting the R-SVC and L-SVC (Figure 11).

**Figure 12:**
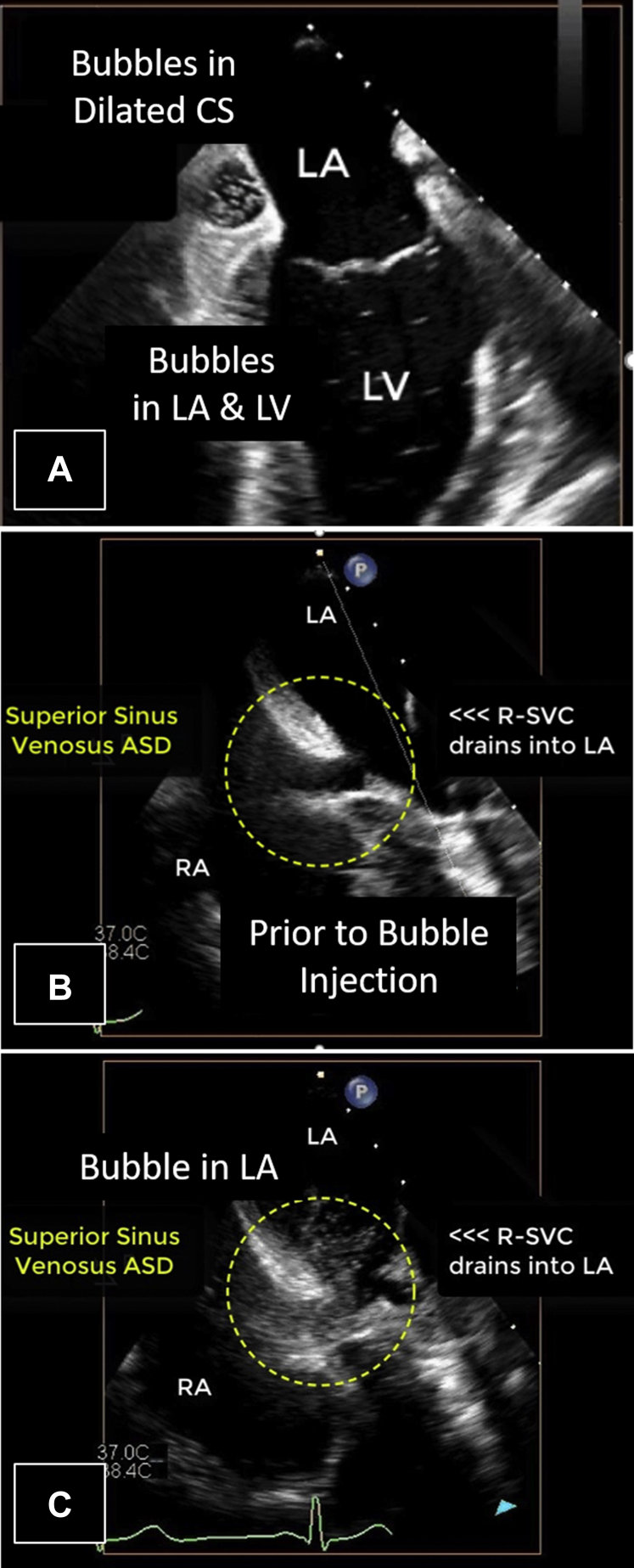
Case 5: R-SVC draining into the LA. Transesophageal echocardiogram with midesophageal view at 90° in grayscale **(A)** and midesophageal view at 115° in grayscale **(B, C)** with intravenous agitated saline injection in the right arm. After injection of agitated saline in the right arm, there is highly unusual pattern of opacification. The dilated CS opacified first, followed quickly by the left heart **(A)**. Video 11 illustrates this unusual opacification pattern. The R-SVC can be seen straddling the superior sinus venosus ASD and draining into the LA **(B, C)**, confirming the right-to-left shunt. Video 12 illustrates this atypical pattern of venous drainage. *CS*, Coronary sinus.

After agitated saline injection into a right arm vein, the pattern of opacification on TEE imaging was very peculiar. After intravenous injection, bubbles appeared immediately in the dilated coronary sinus and a few beats later in the left heart (Figure 12A, Video 11). Immediate opacification of the coronary sinus after right arm injection implies that there is communication (likely via the bridging vein) from the R-SVC to the persistent L-SVC. Moreover, there was a small (8 mm in diameter) sinus venosus defect with left-to-right shunt visualized on color Doppler and after agitated saline injection. The R-SVC drained anomalously and almost completely into the LA. The R-SVC only partly straddled the small superior sinus venosus defect and only partly drained into the RA via the sinus venosus defect (Figure 12B and 12C, Video 12). This drainage pattern created a net right-to-left shunt (R-SVC to LA; this may have been the risk factor for the brain abscess).

**Figure 13:**
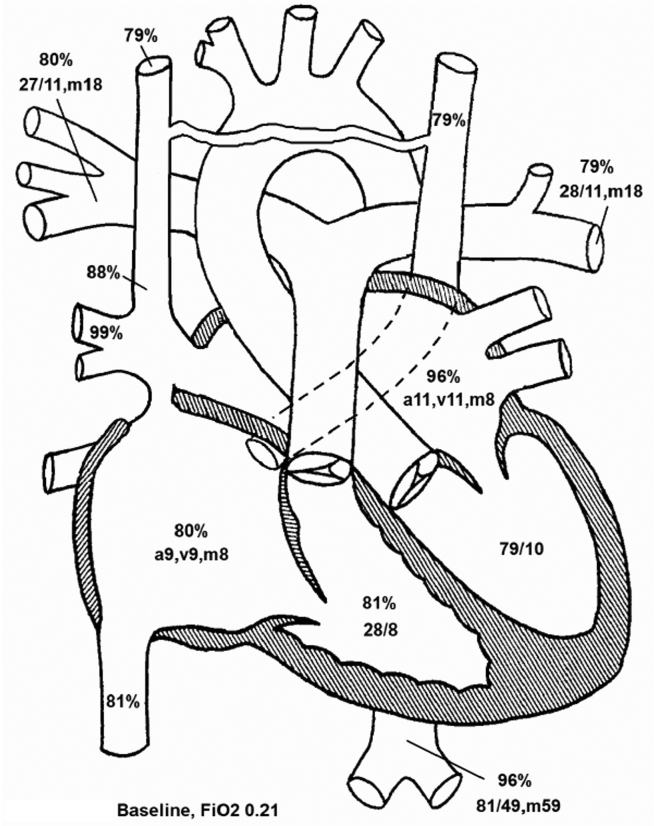
Case 5: R-SVC draining into the LA. Cardiac catheterization diagram with oxygen saturations and chamber pressures. The oxygen saturations and chamber pressures (mm Hg) of each chamber of the heart as determined by cardiac catheterization are represented in this diagram. Atrial chamber pressures are presented in terms of the atrial waveform, and ventricular pressures are presented in terms of systole and diastole. The left-to-right shunt appears insignificant, as blood in the RA is not significantly more oxygenated (80%) than blood returning from the systemic circulation (79%). This was confirmed by determination of the Qp:Qs as 1.2:1, leading to the conclusion that the right-to-left shunt was dominant in this patient. *a*, a-wave pressure; *FiO2*, fraction inspired oxygen; *m*, mean pressure; *v*, v-wave pressure.

Because the R-SVC preferentially drained into the LA and then into a less compliant LV, the flow from the proximal R-SVC was mostly diverted via the innominate vein into the L-SVC. This was because the L-SVC and coronary sinus drained into the right heart, which has higher compliance than the LV. Thus, in this patient, the systemic venous flow was preferentially driven along the following pathway: R-SVC >> bridging vein >> L-SVC >> dilated coronary sinus >> RA >> compliant right ventricle. The remainder of the proximal R-SVC flow was shunted across the sinus venosus defect into the RA (left-to-right shunt). Because the right heart was not significantly dilated, the left-to-right shunt was deemed by TEE to be small. This was confirmed by CMR and cardiac catheterization (Qp:Qs, 1.2 to 1; Figure 13).

Following resolution of his neurosurgical and infectious complications, the patient underwent surgical repair including a Warden procedure, which involves transecting the SVC, connecting the proximal SVC to the right atrial appendage, and redirecting anomalous pulmonary venous flow to the LA by using a patch.^3^

## Discussion

In this case series, we present a variety of thoracic venous anomalies ranging from relatively common to exceedingly rare. Cases 1, 2, and 3 demonstrate the persistent L-SVC, which has a prevalence of 0.3% to 0.5% in the general population and an even higher prevalence among those with other congenital cardiac anomalies.^4^^,^^5^ Cases 3 and 4 demonstrate the unroofed coronary sinus, which is far less common, accounting for only 1% of all ASDs. The combination of persistent L-SVC and an unroofed (or absent) coronary sinus (referred to as Raghib syndrome; case 3) is extremely rare and has only been identified in case reports.^6^, ^7^, ^8^ Case 5 demonstrates PAPVR, which has an estimated prevalence of 0.1%, and a sinus venosus defect, which can co-occur with right upper lobe PAPVR.^9^ However, the atypical anatomy and unusual pattern of venous drainage seen in case 5 appear unique, as there are no identical cases in the literature.

The complexity of cardinal venous system development presents multiple junctures for anomalous development. If, for example, the brachiocephalic anastomosis grows from the right anterior cardinal vein toward the left, an L-SVC may form. Alternatively, if the left anterior and common cardinal veins fail to regress, an L-SVC may persist alongside an R-SVC. Similarly, failed involutions or persistent connections between venous structures may give rise to other patterns of venous drainage, like those presented in cases 3, 4, and 5.

Since persistent L-SVC does not necessitate the presence of a shunt, it usually remains asymptomatic and is often diagnosed incidentally, as in cases 1 and 2. It is classically identified after demonstration of an unusually dilated coronary sinus on echocardiography. The diagnosis of persistent L-SVC is then confirmed after intravenous injection of agitated saline in the left arm demonstrates an abnormal opacification pattern (coronary sinus first, right heart next). Cardiac CT and CMR may provide greater anatomic detail as follow-up studies, although persistent L-SVC may also be identified when those imaging modalities are ordered for other indications.

If the anomalous venous pattern creates a shunt, however, it may present with symptoms of hypoxia or sequelae of paradoxical emboli due to a right-to-left shunt, as in cases 3 and 5. In these cases, identification of the venous anomalies is crucial in determining the etiology of the presenting symptoms. A dilated coronary sinus visualized by echocardiography is usually the salient finding, although CCT and CMR are often required for further characterization of the venous anatomy.

In addition to the consequences of an undesirable venous shunt, there are several important clinical implications for these anomalous venous patterns, even for those categorized as normal variants. Most notably, the presence of a persistent L-SVC or unroofed coronary sinus may significantly impact pacemaker placement, retrograde cardioplegia, or central venous catheter placement. Furthermore, abnormalities of the SVC are associated with an increased risk of abnormalities of the cardiac conduction system, since progenitor pacemaker cells originate near the site of primitive cardinal venous tissue.^10^ As such, it is important for physicians to be able to identify these anatomic variants so patients can be provided with the appropriate imaging, referrals, and interventions without unnecessary errors.

In conclusion, our case series highlights the variability of anomalies arising from the cardinal venous system and the importance of various imaging modalities in identifying those anomalies. Some alternative patterns of venous drainage can be characterized as normal variants, while others can cause pathologic shunting requiring intervention. Since uncomplicated persistent L-SVC is a normal variant, no intervention is needed to correct the venous anomaly, but the knowledge of the alternative pathway is very important when placing catheters or pacing leads. Patterns of venous drainage that create shunting, however, require surgical intervention.^11^

## Supplementary Data

Video 1 Case 1: persistent L-SVC. Transthoracic echocardiogram in the parasternal long-axis view with intravenous agitated saline injection in the left arm. Transthoracic echocardiogram clip demonstrating a dilated CS and atypical opacification pattern after agitated saline injection into the left arm. The dilated CS opacifies first, followed by the right heart. This opacification pattern following agitated saline injection in the left arm is diagnostic of an L-SVC. This video corresponds to Figure 2. *Ao*, Aorta; *CS*, coronary sinus.

Video 2 Case 2: persistent L-SVC, absent R-SVC. Composite transthoracic echocardiogram clip in parasternal long-axis (part 1 of the clip) and modified apical 4-chamber (part 2 of the clip) views with intravenous agitated saline injection in left arm. Transthoracic echocardiogram clip with parasternal long-axis (part 1) and modified apical 4-chamber (part 2) views demonstrating a dilated CS and atypical opacification pattern after agitated saline injection in the left arm. The dilated CS opacifies first, followed by the right heart. As in case 1, this opacification pattern following agitated saline injection in the left arm is diagnostic of an L-SVC. This video corresponds to Figure 3. *Ao*, Aorta; *CS*, coronary sinus.

Video 3 Case 2: persistent L-SVC, absent R-SVC. Transthoracic echocardiogram in parasternal long-axis (PLAX) and modified apical 4-chamber (A4C) views with intravenous agitated saline injection in right arm. Transthoracic echocardiogram clip with parasternal long axis and modified A4C views demonstrating a dilated CS and atypical opacification pattern after agitated saline injection in the right arm. The dilated CS opacifies first, followed by the right heart. When this opacification pattern is observed following agitated saline injection in the right arm, it suggests the absence of the normal R-SVC. This video corresponds to Figure 3. *Ao*, Aorta; *CS*, coronary sinus.

Video 4 Case 3: persistent L-SVC, unroofed CS. Transthoracic echocardiogram in parasternal long-axis view with intravenous agitated saline injection in the left arm. Transthoracic echocardiogram clip demonstrating an unroofed CS and atypical opacification pattern after agitated saline injection into the left arm. The unroofed CS opacifies first, followed by the left heart and then the right heart. This opacification pattern following agitated saline injection in the left arm is diagnostic of both an L-SVC and unroofed CS, also known as Raghib syndrome. This video corresponds to Figure 4. *AV*, Aortic valve; *CS*, coronary sinus.

Video 5 Case 4: unroofed CS without persistent L-SVC. Transthoracic echocardiogram in parasternal long-axis view with unremarkable CS. Despite the identification of an unroofed CS on CCT imaging, the proximal segment of the CS visualized by TTE appears normal and nondilated. This video corresponds to Figure 6. *AV*, Aortic valve; *CS*, coronary sinus.

Video 6 Case 5: R-SVC draining into the LA. Transesophageal echocardiogram with midesophageal view at 0° in grayscale showing dilated coronary sinus. This TEE clip corresponds with Figure 8A and illustrates dilation of the coronary sinus. A dilated coronary sinus suggests abnormally increased blood flow through the coronary sinus as a result of an atypical pattern of venous drainage, such as an L-SVC.

Video 7 Case 5: R-SVC draining into the LA. Transesophageal echocardiogram with midesophageal view at 77° with color Doppler showing dilated coronary sinus. This TEE clip corresponds with Figure 8B and illustrates dilation of and increased blood flow through the coronary sinus seen using color Doppler. Again, these findings indicate an atypical pattern of venous drainage, including an L-SVC.

Video 8 Case 5: R-SVC draining into the LA. Three-dimensional TEE with a zoom image in the so-called surgical view of the mitral valve showing dilated coronary sinus. This TEE clip corresponds with Figure 8C and visualizes the dilated coronary sinus using three-dimensional echocardiography with zoom acquisition and displayed in the so-called surgical view of the mitral valve. *AV*, Aortic valve; *MV*, mitral valve; *TV*, tricuspid valve.

Video 9 Case 5: R-SVC draining into the LA. Transesophageal echocardiogram with midesophageal view at 118° in grayscale showing unusual position of R-SVC. This TEE clip corresponds with Figure 10B and visualizes the atypical position of this patient's R-SVC. The R-SVC usually drains into the RA; however, as shown in this video, the R-SVC in this patient communicates with and drains into the LA. This creates the potential for a right-to-left shunt and may have been the risk factor for this patient's temporal abscess.

Video 10 Case 5: R-SVC draining into the LA. Transesophageal echocardiogram with midesophageal view at 118° with color Doppler showing unusual position of R-SVC. This TEE with color Doppler clip corresponds with Figure 10C and visualizes the communication between the R-SVC and LA. This pattern of venous drainage is highly unusual and increases the risk of sequelae from a right-to-left shunt.

Video 11 Case 5: R-SVC draining into the LA. Simultaneous biplane TEE with midesophageal view at 0° (*left panel)* and 90° *(right panel)* with intravenous agitated saline injection in right arm. This TEE clip corresponds with Figure 12A and demonstrates the atypical opacification pattern observed following injection of agitated saline into the patient's right arm. The dilated coronary sinus opacifies first, followed quickly by the left heart. This suggests communication between the R-SVC and the L-SVC and coronary sinus, likely through the connecting vein seen on CMR (Figure 12).

Video 12 Case 5: R-SVC draining into the LA. Transesophageal echocardiogram with midesophageal view at 115° with intravenous agitated saline injection in the right arm. This TEE clip corresponds with Figure 12B and 12C and demonstrates the abnormal pattern of venous drainage from the R-SVC. Bubbles are seen entering the LA from the R-SVC, traversing the ASD, and then entering the RA. This highly unusual pattern of venous drainage creates a right-to-left shunt that predisposed this patient to a brain abscess.
